# Lunar Lithium-7 Sensing (*δ*7Li): Spectral Patterns and Artificial Intelligence Techniques

**DOI:** 10.3390/s24123931

**Published:** 2024-06-17

**Authors:** Julia Fernandez, Susana Fernandez, Enrique Diez, Noemi Pinilla-Alonso, Saúl Pérez, Santiago Iglesias, Alejandro Buendía, Javier Rodríguez, Javier de Cos

**Affiliations:** 1 Instituto de Ciencias y Tecnologías Espaciales de Asturias, Universidad de Oviedo, 33004 Oviedo, Spain; fernandezmsusana@uniovi.es (S.F.); diezenrique@uniovi.es (E.D.); perezsaul@uniovi.es (S.P.); iglesiassantiago@uniovi.es (S.I.); buendiaalejandro@uniovi.es (A.B.); uo204336@uniovi.es (J.R.); 2 Florida Space Institute, University of Central Florida, Orlando, FL 32816, USA; npinilla@ucf.edu

**Keywords:** Lithium-7, bootstrapping, regression model, spectral patterns, artificial neural networks, MARS, 68T05, 68T07, 85-08

## Abstract

Lithium, a critical natural resource integral to modern technology, has influenced diverse industries since its discovery in the 1950s. Of particular interest is lithium-7, the most prevalent lithium isotope on Earth, playing a vital role in applications such as batteries, metal alloys, medicine, and nuclear research. However, its extraction presents significant environmental and logistical challenges. This article explores the potential for lithium exploration on the Moon, driven by its value as a resource and the prospect of cost reduction due to the Moon’s lower gravity, which holds promise for future space exploration endeavors. Additionally, the presence of lithium in the solar wind and its implications for material transport across celestial bodies are subjects of intrigue. Drawing from a limited dataset collected during the Apollo missions (Apollo 12, 15, 16, and 17) and leveraging artificial intelligence techniques and sample expansion through bootstrapping, this study develops predictive models for lithium-7 concentration based on spectral patterns. The study areas encompass the Aitken crater, Hadley Rima, and the Taurus–Littrow Valley, where higher lithium concentrations are observed in basaltic lunar regions. This research bridges lunar geology and the formation of the solar system, providing valuable insights into celestial resources and enhancing our understanding of space. The data used in this study were obtained from the imaging sensors (infrared, visible, and ultraviolet) of the Clementine satellite, which significantly contributed to the success of our research. Furthermore, the study addresses various aspects related to statistical analysis, sample quality validation, resampling, and bootstrapping. Supervised machine learning model training and validation, as well as data import and export, were explored. The analysis of data generated by the Clementine probe in the near-infrared (NIR) and ultraviolet-visible (UVVIS) spectra revealed evidence of the presence of lithium-7 (Li-7) on the lunar surface. The distribution of Li-7 on the lunar surface is non-uniform, with varying concentrations in different regions of the Moon identified, supporting the initial hypothesis associating surface Li-7 concentration with exposure to solar wind. While a direct numerical relationship between lunar topography and Li-7 concentration has not been established due to morphological diversity and methodological limitations, preliminary results suggest significant economic and technological potential in lunar lithium exploration and extraction.

## 1. Introduction

In the context of modern technology, lithium holds a pivotal role as a critical natural resource. Large lithium deposits were discovered and developed in South America, particularly in countries such as Bolivia, Chile, and Argentina, during the mid-20th century. The utilization of lithium has transformed and revolutionized diverse industries. By the 1970s, the production of lithium batteries commenced, opening new frontiers in technological advancements [1].

Lithium-7, the most abundant isotope of lithium, constitutes approximately 92.5% of all lithium found on Earth. Known for its lightweight and low-density properties, lithium-7 is essential in various applications, including nuclear research, battery technology, metal alloys, and medical treatments [1,2,3,4]. While natural lithium is widely used in many of these applications, our study specifically focuses on detecting and analyzing the lithium-7 isotope.

The extraction and production processes of lithium can consume large amounts of water and energy, potentially leading to environmental concerns such as soil and water pollution. Additionally, the growing demand for lithium-ion batteries may result in supply issues and increased costs [3].

Although the extraction of lithium on the Moon is not currently practical due to technical and logistical challenges, it remains a future possibility thanks to ongoing research and the development of advanced space technologies [4]. Exploring the potential extraction of lithium on the Moon could provide valuable insights into lunar geology and the formation of the solar system.

Another reason for the interest in lunar lithium exploration is that lithium is a relatively lightweight element but is found in small quantities on Earth, making its extraction expensive. While the Moon’s approximately six times lower gravity than Earth could theoretically reduce the cost of transporting lunar-extracted lithium to Earth, the overall cost is expected to be prohibitive compared to terrestrial transport unless exceptionally rich lithium deposits are discovered. Consequently, the primary focus may be on the in situ utilization of lunar resources, which also holds promise for future exploration and potential exploitation of similar resources on other satellites and planets [5,6].

The presence of lithium in the solar wind, a continuous stream of charged particles, is of interest for space exploration and scientific research. It is believed that the solar wind can transport materials from the Sun to Earth and other solar system bodies, including the Moon. Studying the composition of the solar wind is essential for gaining a better understanding of solar processes and their effects on planets and satellites.

The exploration for lithium in various lunar geological formations, including craters, stems from the hypothesis that these formations might contain deposits rich in lithium-bearing minerals. Notably, spodumene, a significant source of lithium on Earth [7].

Exploration of the Moon and the collection of rock and regolith samples began in the 1960s and early 1970s as part of various space programs. Lithium had already been detected in lunar samples from earlier Apollo missions, such as Apollo 12, Apollo 15, and Apollo 16, which, respectively, took place in 1969, 1971, and 1972, providing some of the initial evidence of lunar lithium presence. The comprehensive analysis of lithium in lunar samples continued with subsequent missions, including Apollo 17 in 1972, which further contributed to our understanding of lunar resources.

The NASA Clementine spacecraft mission, launched in 1994, was a joint effort between NASA and the U.S. Department of Defense to map the lunar surface and conduct research on the presence of various elements [8]. The “Clementine On-Line data Volumes” provide valuable data from NASA, including lightweight and high-resolution images in separate ultraviolet, blue, green, red, and infrared bands.

Although previous mapping and detection missions to the Moon could detect spectral indications of lithium, none of them yielded conclusive data regarding its existence. Therefore, to conduct the proposed analyses in this study, data from samples collected during the Apollo 11, 12, 14, 16, and 17 missions were available, including precise information about their lithium and lithium-7 contents and the coordinates defining the exact points from which they were extracted.

The Apollo missions were meticulously planned and executed to ensure the safe collection, handling, and analysis of lunar samples. Following the landing of Apollo 11 in 1969, a total of 382 kg of lunar rocks and soil, consisting of 2196 individual specimens, were brought back to Earth by the six Apollo missions. The Interagency Committee on Back Contamination (ICBC) oversaw quarantine protocols to prevent potential contamination of Earth’s biosphere, although the likelihood of lunar microbes was considered low [9].

The samples were initially processed in the Lunar Receiving Laboratory (LRL) at the Manned Spacecraft Center, now known as the Johnson Space Center. The LRL was equipped with state-of-the-art facilities, including high vacuum environments and nitrogen-filled glove boxes to minimize terrestrial contamination and preserve lunar conditions. Biological quarantine protocols were strictly followed during the Apollo 11, 12, and 14 missions, consuming only 2% of the samples for these tests. The subsequent missions continued with biomedical follow-up testing without the need for quarantine [9].

To address concerns about contamination, a portion of the samples was permanently stored under vacuum conditions, and a remote storage facility was established to safeguard a part of the collection. The current curation practices at the Johnson Space Center focus on maintaining sample purity, preserving historical information, and facilitating ongoing research and education [9].

Drawing parallels to our study on lunar Li-7 analysis, the utilization of both infrared and UVVIS spectroscopic techniques holds immense potential for enhancing our understanding of lunar regolith composition and evolution. However, to bridge the gap between the remote sensing data obtained from orbiters and the ground truth data derived from physical moon rock samples, it is imperative to establish a correlation between these datasets. The analysis of physical samples on Earth provides invaluable ground truth data that can be correlated with the remote sensing data obtained from orbiters. By precisely determining the composition and original location of these samples, we can correlate the actual concentrations of lithium and lithium-7 measured in the extracted samples with their emission spectra obtained from orbital scans. This correlation enhances the accuracy and reliability of our analysis and contributes to a deeper understanding of lunar geology and resource distribution.

In recent years, the application of spectroscopic techniques spanning both the infrared (IR) and ultraviolet/visible (UVVIS) ranges has garnered significant attention in the field of geology research, offering valuable insights into mineralogical composition and alteration processes. These technologies present a powerful toolset for characterizing geological features and understanding complex phenomena occurring both on Earth and beyond.

Infrared spectroscopy, particularly in the short-wavelength infrared (SWIR) range, has emerged as a promising method for mapping mineralogical variations in geological formations. Studies have demonstrated its efficacy in identifying mineral assemblages and delineating alteration zones associated with ore deposition [10]. Additionally, ultraviolet/visible (UVVIS) spectroscopy provides complementary information, capturing variations in optical properties that can further elucidate geological characteristics.

Similarly, advancements in infrared holographic detection have revolutionized the observation and analysis of crystallization processes, enabling researchers to capture dynamic phase attributes with unprecedented detail [11]. By combining a free field of view scheme with infrared holography, researchers have been able to observe phase transitions and crystal growth in real-time, shedding light on the fundamental principles governing crystal formation and behavior.

Drawing parallels to our study on lunar Li-7 analysis, the utilization of both infrared and UVVIS spectroscopic techniques holds immense potential for enhancing our understanding of lunar regolith composition and evolution.

The aim is to develop a regression model capable of estimating lithium and lithium-7 concentrations in each lunar area based on spectral patterns. Due to the limited number of available samples and the goal of developing more accurate models using artificial intelligence, bootstrapping techniques are employed to expand the sample population while maintaining statistical integrity. By leveraging the unique capabilities of these spectroscopic methods, we aim to extract valuable insights into the distribution and characteristics of Li-7 isotopes on the lunar surface. This endeavor not only contributes to our knowledge of lunar geology but also underscores the broader significance of spectroscopic technologies in planetary exploration and resource assessment.

Once an expanded sample is available, two distinct artificial intelligence techniques are employed to generate models capable of predicting lithium and lithium-7 concentrations in each lunar area based on their spectral values.

To optimize the identification of sought-after lithium concentrations, different training areas are studied, including the Aitken crater, Hadley Rima, and a specific land site, the Taurus–Littrow Valley, where a higher concentration of lithium is observed in basaltic regions in the Moon’s lower-lying areas.

## 2. Materials and Methods

Upon acquiring actual concentration data of Li-7 from precisely determined and geolocated zones on the lunar surface, derived from the detailed analysis of various samples collected during the different Apollo missions, the utilization of tools enabling the extraction of spectrographic information from various public geoportals becomes imperative. Subsequently, this extracted information is implemented into a cartographic model, forming an integral aspect of our study. This task involves a verification effort of value ranges and the utilization of digital elevation models extracted from the LOLA instrument [12], as well as images from the visible (VIS) and near-infrared (NIR) sensors of the Clementine mission [13]. Additionally, soil samples from the Apollo missions were obtained from various geoportals, as detailed below.

### 2.1. Geospatial Databases

**Orbital Data Explorer** is a tool within NASA’s Planetary Data System Geosciences Node that allows for the query, search, visualization, and retrieval of orbital information from various missions utilizing different instruments that have collected extensive data from Mars, Mercury, Venus, and the Earth’s Moon [14].

**PILOT** (Planetary Image Locator) is a planetary image location tool belonging to the Cartography and Imaging Sciences Discipline Node of NASA’s Planetary Data System, where the primary collections of digital images from past, present, and future planetary missions are integrated. The system provides the planetary science community at NASA with digital image archives, auxiliary data, sophisticated data search and retrieval tools, as well as the cartographic and technical expertise necessary to fully utilize the extensive collection of digital planetary images from many terrestrial planetary bodies, including icy satellites [15].

**Astropedia** is a planetary and lunar cartographic catalog that allows for the selection of different missions and instruments for obtaining information about a specific study area. These data have facilitated the discovery of relatively small lunar features, enabled the determination of their relative ages, and supported studies such as crater counting, geological mapping, structural analysis, site characterization, and others, which contribute to the understanding of the lunar geological record. Low-sunlight and double-illumination topographic and cartographic (TC) data provide morphological information that supports geological mapping. The availability of coverages in multiple illuminations ensures that any site of interest can be studied [16].

**QGIS** is a Geographic Information System (GIS) application that allows for map composition and spatial data exploration, facilitating the visualization, management, editing, and analysis of georeferenced information from databases, vector, and raster images [17].

### 2.2. Instrumentation

**Near-Infrared Mapping Spectrometer (NIMS)**

The near-infrared mapping spectrometer (NIMS) aboard the Clementine mission played a pivotal role in capturing detailed data on the lunar surface [18]. Key technical specifications of the NIMS include:

- **Wavelength Range:** 0.7 to 3.1 μm
- **Spectral Resolution:** Variable, depending on the operational mode
- **Spatial Resolution:** Approximately 1.5 km/pixel
- **Operational Mode:** Provided both imaging and spectroscopic capabilities
- **Purpose:** Facilitated the analysis of mineral composition on the lunar surface through near-infrared spectroscopy.

The NIMS camera operated during the Clementine mission, offering a valuable dataset for our study.

**Ultraviolet/Visible Imaging Spectrometer (UVVIS)**

The ultraviolet/visible imaging spectrometer (UVVIS) was another instrumental component of the Clementine mission, designed to capture data across ultraviolet and visible wavelengths [19]. The essential technical details include the following:

- **Wavelength Range:** Ultraviolet to visible spectrum, covering approximately 0.25 to 1.1 μm
- **Spectral Resolution:** Varies across the ultraviolet and visible bands
- **Spatial Resolution:** Approximately 0.1 to 0.15 km/pixel
- **Operational Mode:** Provided imaging and spectroscopic capabilities
- **Purpose:** Enabled the collection of data on optical properties, distribution of chemical elements, and geological processes on the lunar surface.

To ensure consistent spatial alignment and integration of data from the NIR and UVVIS sensors, the Clementine mission employed rigorous geometric processing. Both the NIR and UVVIS datasets were geometrically controlled to the 750-nm Clementine basemap mosaic. This base map provided a common pixel size and was produced using the USGS integrated software for imagers and spectrometers (ISIS 3.6.0). The processing steps included radiometric correction, geometric control, spectral registration, photometric normalization, and image mosaicking. The NIR spectral bands were coregistered to a precision of 0.2 pixels, ensuring accurate superposition with the UVVIS data. Photometric normalization was applied to balance brightness variations due to different illumination conditions, resulting in near-seamless, uniformly illuminated views of the lunar surface.

These technical specifications highlight the capabilities of both the NIMS and UVVIS cameras, crucial instruments in our investigation of the lunar surface composition and characteristics.

### 2.3. Statistical Software and Modeling Methods

In this study, the selection of the R programming language and the RStudio development environment was deliberate, driven by their tailored suitability for the intricate statistical and spatial analyses required. R, renowned for its versatility and extensive library of packages, emerged as the optimal choice due to its robust capabilities in statistical analysis and mathematical modeling within the realm of data science [20,21]. With its interactive command execution and vast package collection, R facilitated the implementation of sophisticated models and seamless integration with diverse datasets, thereby enabling rigorous statistical analyses essential for scientific investigations.

Complementing R, the utilization of the dedicated integrated development environment (IDE), RStudio, streamlined our programming tasks and enhanced efficiency. Its intuitive tools for project organization and code development, coupled with advanced visualization features, provided a comprehensive statistical analysis environment conducive to our research goals.

Specifically, the combined use of R and RStudio proved pivotal for analyzing the geospatial data imported from QGIS, a critical aspect of our study. This integration facilitated seamless collaboration between statistical and spatial analyses, aligning closely with the intricate requirements of our research objectives. Furthermore, the effective importation of geospatial data from QGIS into our R environment underscored the synergy between statistical and spatial analyses, empowering us to derive meaningful insights from the data.

The **multivariate adaptive regression model (MARS)** introduced by Friedman [22] is a classification or multivariate nonparametric regression technique. Its primary aim is to predict the values of a continuous dependent variable, denoted as y (n × 1), based on a set of independent explanatory variables, X (n × p). The MARS model can be expressed as follows:

(1) $$y=f\left(X\right)+e\left(6\right)$$

Here, ‘*f*’ represents the sum of the base functions, and ‘*e*’ is an error vector with dimensions (n × 1).

One of the significant advantages of the MARS model is its lack of a priori assumptions about the functional relationship between the dependent and independent variables. This relationship is modeled using a subset of coefficients and piecewise linear splines (basic functions). These basic functions span the space of q-th order spline approximation, fitting the basis function coefficients to the data through ordinary least-squares (14). In the case of a univariate scenario (n = 1) with K + 1 regions defined by K points on the real line (knots), one such basis is represented by the following functions [22]:

(2) $$\left[-{\left(x-t\right)}_{+}^{q}\right]=\left\{\begin{array}{cc} \; & \;\mathrm{if}\;x<t,\;{\left(t-x\right)}^{q}\\ \; & \;\mathrm{Otherwise}\;,\;\;\;0\; \end{array}\right.$$

(3) $$\left[+{\left(x-t\right)}_{+}^{q}\right]=\left\{\begin{array}{cc} \; & \;\mathrm{if}\;x\geq t,\;{\left(t-x\right)}^{q}\\ \; & \;\mathrm{Otherwise}\;,\;\;\;0\; \end{array}\right.$$

where ‘*q*’ (≥0) represents the power of the basis functions. The MARS model for a dependent variable y with M basis functions (terms) can be described as

(4) $$\hat{y}=\hat{f_{M}}\left(x\right)=c_{0}+\sum_{m=1}^{M}c_{m}B_{m}\left(x\right)$$

Here, $\hat{y}$ is the dependent variable predicted by the MARS model, $c_{0}$ is a constant, *B_m_*(*x*) is the mth basis function, and $c_{m}$ is the coefficient of the *m*-th basis function. Both variables entered in the model as the positions of the nodes for each individual variable must be optimized. For each data set X, containing n objects and p explanatory variables, the final model is constructed using a two-step approach.

Firstly, to select the consecutive base pairs of the model functions, a two-step forward method is implemented. This progressive selection of basis functions can lead to a highly complex and overfitted model. While such a model fits the data well, it often lacks predictive ability for new objects. To improve prediction, redundant base functions are removed one at a time using a regression procedure. MARS uses the generalized cross validation (*GCV*) to determine which basis functions are included in the model.

The GCV criterion is defined as follows [23]:

(5) $$GVC\left(M\right)=\frac{\frac{1}{n}\sum_{i=1}^{n}{\left(y_{i}-\hat{f_{M}\left(x_{i}\right)}\right)}^{2}}{{\left(1-\frac{C(m)}{n}\right)}^{2}}$$

Here, *C*(*M*) represents the penalty due to the model’s complexity, which increases with the number of basis functions. The model is defined as

(6) C(m)=(M+1)+dM

where ’*M*’ is the number of basis functions, and ’*d*’ is a penalty parameter for each base function included in the model. In the present study, the parameter ’*d*’ is set to 2, and the maximum number of tracer interaction type base functions is restricted to 3.

**Figure 1:**
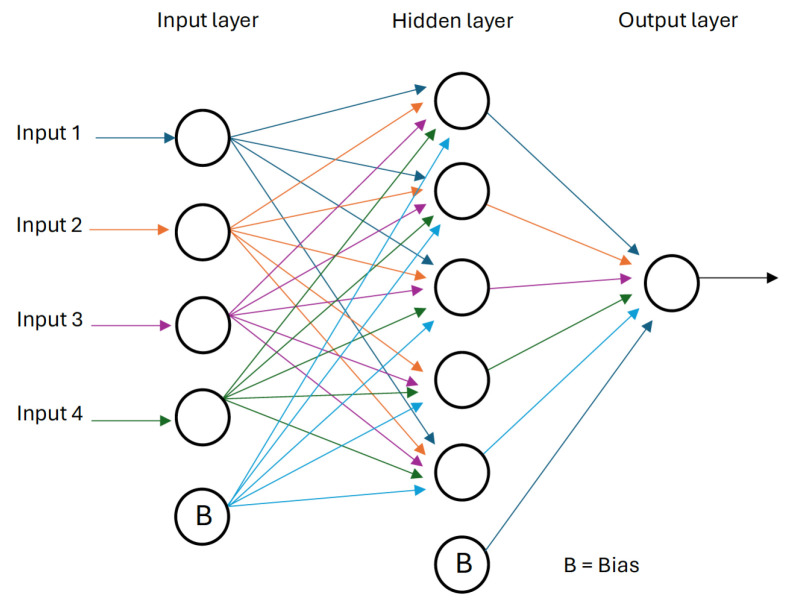
Feedforward MLP neural network.

**Artificial neural networks** are computational models inspired by biological neural networks that consist of a series of interconnected simple processing elements called neurons or nodes [24] . As it is well-known, one of the main advantages of neural networks lays in their ability to represent both linear and non-linear models by learning directly from data measurements [25]. The multi-layer perceptron is a specific type of feedforward neural network. The nodes are organized in layers (input, hidden, and output layers), and each neuron relates to one or more nodes of the following layers only. There is a special type of node called “bias” that has no connection with the neurons in the previous layers. These are used to shift the y-intercept value of the activation function for the next layers and, therefore, enhance the flexibility of the network. Figure 1 shows a scheme of an MLP neural network.

Every neuron receives a stream of input data from either the neurons in the preceding layer or an external source. The neuron then locally processes these data using an activation or transfer function and transmits the outcome to one or more nodes within any of the subsequent layers. This process continues iteratively until the output neurons are reached. Each connection linking neurons is assigned a numerical value that signifies the significance of the preceding neuron’s contribution to the current one, referred to as “synaptic weight” or simply “weight”. It is within these values that the most critical part of knowledge is encapsulated [26]. From a mathematical standpoint, a neuron within the MLP neural network can be mathematically represented as

(7) $$Z_{j}\left(t\right)=g\left(S_{i}\right)=g\left(\sum_{i=1}^{n}W_{ji}.Z_{i}\left(t-1\right)+W_{j}\right)$$

where *Z_j_* represents the output of the *j*th neuron, *W_ji_* denotes the synaptic weight connecting the *j*th and *i*th neurons, *t* signifies the current layer of the *Z_j_* neuron, *Z_i_* stands for the output of the ith node from the preceding (*t* − 1) layer, and *W_j_* corresponds to the bias term. The function *g*(·) is commonly referred to as the activation function, responsible for the local transformation of the input.

In supervised learning, the training process involves providing a well-chosen set of input–output pairs. The weights are adjusted to adapt to the underlying data patterns. The way weights are altered to achieve the desired outcome is known as the “learning algorithm”, and it is a pivotal element influencing the neural network’s performance [27]. The backpropagation error is a widely utilized learning algorithm in the realm of neural networks. It operates by minimizing the squared disparity between the target value and the anticipated output across all input–output pairs. The calculation of the squared error can be expressed as

(8) $$E=\frac{1}{2}\sum i\sum j{\left(y_{ij}-d_{ij}\right)}^{2}$$

In this context, where *y_ij_* represents the predicted net value, *d_ij_* stands for the target value, *i* ranges from 1 to the total number of input–output pairs, and *j* is an index corresponding to each output node. To achieve the minimization of *E* through gradient descent, it becomes imperative to calculate the partial derivative of E concerning each weight:

(9) $$\Delta W_{ij}=-\frac{\partial E}{\partial x_{i}}.\frac{\partial x_{i}}{\partial w_{ij}}=\frac{\partial E}{\partial x_{i}}.\frac{\partial }{\partial w_{ij}}\sum_{k}w_{ik}.y_{k}=\delta_{i}w_{ij}$$

Using *W_ij_* to establish a connection between the *i*th and *j*th neurons, we need to calculate errors ($\delta_{i}$) for all neurons within the network. This process can be efficiently achieved in two distinct steps:

In the first step, we calculate the state of all neurons, including the hidden ones, using Equation (7).

In the second step, we propagate the errors backward, ultimately reaching the input units. By employing the chain rule, we can determine the errors of the hidden units by expressing them in relation to the states, errors, and weights of their subsequent layer nodes, as illustrated in Equation (10):

(10) $$\delta_{i}=\frac{\partial E}{\partial x_{i}}=-\frac{\partial E}{\partial x_{i}}.\frac{\partial x_{i}}{\partial y_{j}}.\frac{\partial y_{j}}{\partial x_{i}}=f^{\prime }\left(x_{j}\right).\sum_{i}\delta_{i}.w_{ij}$$

With the knowledge of the error in the output nodes, we can determine the errors in the hidden layers that precede them and continue this process until we reach the input neurons. This enables us to calculate the negative error gradient for all units within the network.

Typically, the weights are adjusted in a manner proportional to the negative gradient calculated using this method. The proportional factor is referred to as the “learning rate” and is a crucial parameter in the training of neural networks [26]:

(11) $$\Delta w_{ij}=-\varepsilon \frac{\partial E}{\partial w_{ij}}$$

There are two approaches to utilizing the algorithm: one involves adjusting the weights after each input–output pair, which minimizes the required memory for the algorithm’s operation. The other approach, as applied in this study, involves accumulating all the weight gradients for all the data pairs before implementing the changes. This process of computing weight adjustments for all the data pairs is repeated several times, as specified by the user. Each such iteration is termed an “Epoch”.

It is essential to exercise caution to prevent overtraining. The speed of learning and the magnitude of weight changes in neural networks are influenced by the learning rate. If the interplay between epochs, learning rate, and data quality is not well-balanced, the network can become either undertrained or overtrained, leading to a decline in performance.

To assess the performance of the network, we employed three metrics: root mean square error (RMSE), normalized error, and accuracy. RMSE is commonly utilized in regression and similar scenarios, as it quantifies the extent to which the outputs deviate from the desired target values:

(12) $$RMSE=\sqrt{\frac{1}{N}\sum_{i=1}^{N}{\left(y_{i}-d_{i}\right)}^{2}}$$

Here, *N* represents the number of outputs, *y_j_* signifies the predicted values, *d_j_* denotes the target values, and *n* is the number of observations. However, one limitation of RMSE is that it is affected by the variances in the target values, making it unsuitable for direct comparison with neural networks operating in diverse contexts. Variance, in this context, is defined as the average squared deviation from the mean of a variable within a population, as expressed in Equation (13):

(13) $$\mathrm{Variance}=\frac{1}{N}\sum_{i=1}^{N}{\left(d_{i}-\mu \right)}^{2}$$

where *N* represents the number of observations, and μ signifies the mean of the variable. This measure can be applied to the entire network or to each neuron individually, particularly in cases where different variances exist for each node. The normalized error is a metric that mitigates the influence of target variance and remains unaffected by network configurations [28]. It yields values within the range of 0 to 1 and can be regarded as a gauge of output variance resulting from errors, rather than being influenced by target variance or network architecture.

To calculate the normalized error, it is imperative to compute the sum of squared deviations of the target values from their mean, as specified in Equation (14):

(14) $$E_{mean}=\sum_{j=1}^{M}\sum_{i=1}^{N}{\left(d_{ji}-\mu_{j}\right)}^{2}$$

In this context, *N* represents the number of observations, and *M* signifies the number of outputs. The total squared error of the network is calculated as follows:

(15) $$E_{t}=\sum_{j=1}^{M}\sum_{i=1}^{N}{\left(y_{j}-d_{j}\right)}_{i}^{2}$$

Hence, the normalized error is formally defined as

(16) $$E_{n}=\frac{E_{t}}{E_{mean}}$$

A lower value of *E_n_* is preferable, as it signifies that the pattern is being learned effectively. Conversely, an *E_n_* value close to 1 suggests that the network is essentially returning the mean as the desired output for all input sets. Backpropagation neural networks tend to excel in learning such patterns, making the normalized error particularly valuable for them [29]. This metric can be applied either to the entire network or to individual output variables.

Accuracy, on the other hand, is defined as the proportion of correct predictions relative to the dataset’s size. It is commonly utilized in networks with discontinuous outputs, as it is straightforward to calculate for both positive and negative values. However, in the case of continuous outputs, a threshold error value must be set to classify the predicted values and assess the network’s accuracy. In this study, the threshold value is set at 5ª of the full interval for each output [30].

**Description of the Bootstrapping Process**:

The bootstrap standard method with resampling is a statistical technique utilized in this study to estimate the sampling distribution of a statistic from a given dataset. In our analysis, we employed this method to address the challenge of limited data availability, which is common in studies utilizing machine learning techniques [31].

Initially, we utilized a dataset, denoted as X, which contained a total of 8 samples. Each sample represented unique observations obtained from the lunar surface, capturing the concentration of Li-7 and reflectance values at various wavelengths.

The variables included for each sample were as follows:

Concentration of Li-7: The concentration of Li-7 in each sample was measured and recorded as part of the dataset.

Reflectance Values: Reflectance values were measured at multiple wavelengths using data acquired from both the near-infrared mapping spectrometer (NIMS) and ultraviolet/visible imaging spectrometer (UVVIS) cameras employed during the Clementine mission. Specifically, reflectance data were collected for 6 near-infrared (NIR) wavelengths and 5 ultraviolet/visible (UVVIS) wavelengths.

The bootstrapping process involved iterative resampling of the dataset X. Specifically, for each iteration, a new dataset, denoted as $X_{i}$, was created by randomly drawing 8 observations from the original dataset with replacement. This resampling procedure allowed us to generate multiple datasets, each of which captured different variations within the original data.

Next, a statistic of interest, denoted as Theta_i_, was computed for each resampled dataset $X_{i}$. This statistic was calculated using the Formula (17), where $Z_{j}\left(t\right)$ represents the computed statistic for each iteration, $W_{ji}$ and $W_{j}$ are the weights, and $Z_{i}\left(t-1\right)$ represents the values from the previous iteration.

(17) $$Z_{j}\left(t\right)=g\left(S_{i}\right)=g\sum_{i=1}^{n}\left(W_{ji}Z_{i}\left(t-1\right)+W_{j}\right)$$

The collection of these computed statistics, Theta_i_, formed the bootstrap distribution. By analyzing this distribution, we were able to estimate the sampling distribution of the statistic and derive valuable insights into the variability and uncertainty associated with our results.

## 3. Results and Discussion

In our investigation, a comprehensive statistical analysis was conducted on a set of original samples collected during the Apollo 12, 15, 16, and 17 missions. The primary focus of our analysis lies in evaluating the concentration of Li-7, measured in % by weight and ranging from 3.35% to 8.89%. Our examination extends to reflectance values at various wavelengths, with data acquired from both the near-infrared mapping spectrometer (NIMS) and ultraviolet/visible imaging spectrometer (UVVIS) cameras employed during the Clementine mission.

Table 1 provides descriptive statistics of the Li-7 concentration, along with the corresponding reflectance values obtained through the NIMS camera, which operated in the near-infrared range. This camera facilitated a detailed exploration of the mineral composition of the lunar surface. Table 2 contributes insights into the optical properties of the lunar surface, presenting reflectance values obtained via the UVVIS camera during the Clementine mission. This camera, covering ultraviolet and visible wavelengths, played a crucial role in collecting data relevant to discerning the distribution of chemical elements and geological processes on the Moon. The integration of the statistical analyses and data retrieved from both the NIMS and UVVIS cameras offers a comprehensive understanding of the composition and characteristics of the lunar surface. These findings are particularly relevant for investigating geological processes and the presence of chemical elements across multiple samples collected from our natural satellite during different Apollo missions.

Since the available dataset is significantly reduced and not suitable for the application of intelligent supervised learning models, the bootstrap technique, previously explained, was applied to obtain a random sample of 100 data points. The descriptive statistics for this new sample are presented in Table 3 and Table 4.

In the newly generated sample, it is possible to observe that the minimum value in % by weight of the concentration of Li-7 has increased, while the maximum value has decreased, as seen in Table 4. The reflectance values obtained with the NIMS cameras for wavelengths of 415, 750, 900, 950, and 1000, as well as the UVVIS values for wavelengths of 1100, 1250, 1500, 2000, 2600, and 2780 measured in nm, exhibit the same effect. Bootstrapping is a resampling technique used to estimate the distribution of a statistic by generating multiple random samples with replacement from the original sample. By minimizing the influence of outliers and improving the representativeness of the sample, bootstrapping can contribute to reducing the maximum value and increasing the minimum value in the concentration of Li-7, as well as to the effects observed in reflectance measurements and UVVIS values for different wavelengths.

Through the predictive techniques of artificial intelligence corresponding to the multivariate adaptive regression splines (MARS) and artificial neural network (ANN) models, information regarding the concentrations of Li-7 in relation to albedo values was obtained. In the case of the MARS model, both the graphical representation of the model and the variables ranked by reflectivity importance were obtained. It is determined that the Li-7 concentration data are calculated using the values of some of the parameters as if they were of order 2 (Earth package). The training results yield model fit values in terms of R2 that are exceptionally high (around 0.98), which suggests that predicting the concentration of Li-7 based on the available wavelength values is feasible, and a high level of precision can be expected.

For the ANN models, the ’AMORE’ library (a modular regression) in R is utilized, providing functions for constructing, training, and evaluating artificial neural network models. The training process is customized, beginning with a bootstrap of 1000 data points, which are then normalized to a range of −1 to 1 (normalized original dataset).

Following this, an 80% random subsample of the original data is extracted for training the intelligent model, while the remaining 20% is reserved for the final evaluation of the neural network using data not included in the training process. Within the 80% allocated for training, a secondary random subdivision is performed, dividing it into two datasets: one containing 80% for training and the other 20% for internal validation of the training process. In this study, various network architectures were explored, and after thorough experimentation, the configuration of 11 input neurons, 18 hidden neurons, and 2 output neurons was determined as the most suitable for implementing a multi-layer perceptron (MLP) using the AMORE library in R. Several key parameters crucial for MLP training were meticulously adjusted to optimize predictive performance. The global learning rate was set at 10^−3^ to regulate convergence speed, and a fixed global momentum of 0.5 was implemented to enhance training stability. The chosen error criterion was the mean squared error, and the hyperbolic tangent activation function was applied to both the hidden and output layers. The training employed the adaptive gradient descent with momentum algorithm. These adjustments were made in consideration of the specific dataset characteristics and were validated through extensive experimentation.

**Figure 2:**
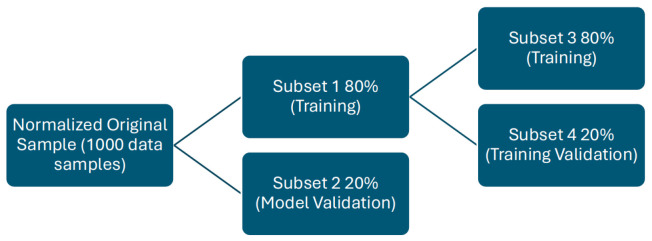
Customizing the Training Process.

After completing the model training, its performance is assessed through a validation process using 20% of the original data, which was specifically reserved for this purpose during the training phase. With the assistance of the “Metrics” package, the mean squared error generated by the neural network when predicting Li-7 concentrations in this subset of data is determined. The result revealed an error of 0.12943, which confirms the effectiveness of the developed multi-layer perceptron (MLP) architecture and its parameter configuration in accurately predicting outcomes, particularly regarding Li-7 concentrations. These results are deemed acceptable for the objectives outlined in this study. (See Figure 2).

### 3.1. Selection of Lunar Study Sites

When selecting the specific locations in which to apply the algorithms developed, it should be considered that the Moon is a celestial body with unique and diverse geological characteristics. However, there are two geological structures of high interest for the object of this study that are particularly suitable for the validation of the intelligent models developed due to both their topographic variability and their formation processes. These structures are the **rilles**, linear and narrow geological structures found on the lunar surface that provide evidence of the satellite’s volcanic history, and **impact craters**, prominent and common geological structures on the lunar surface. They form when an object, such as a meteorite or asteroid, collides with the Moon at high speed. The morphology of these craters can reveal details about the collision energy, velocity, and angle of entry of the impacting object. In addition, the distribution and density of impact craters on the lunar surface are used to estimate the relative age of different lunar regions, as areas with more craters are considered older due to the continuous accumulation of impacts over time. Finally, it is deemed appropriate to apply the algorithms developed at the **Apollo 17 landing site**, where samples were analyzed with values of lithium-7, in order to verify if the data estimated by the models based on the images obtained in the VVIS and NIR fall within the actual ranges.

In our study, the spectral data obtained from infrared and ultraviolet-visible images captured by the Clementine mission and stored in the Orbital Data Explorer database were processed using QGIS 3.30 software. These images were imported into QGIS, and the values for each wavelength of the six infrared bands and the five ultraviolet-visible bands contained in the images were extracted for each pixel. These 11 values were then inputted into the previously trained models, which returned a concentration value of Li-7 for each pixel in the image. Subsequently, these Li-7 concentration values were projected onto elevation models of the study area to visualize their spatial distribution. Additionally, they were correlated with the slopes of the elevation models to investigate any relationship between slope and Li-7 concentration. This analysis aims to validate the hypothesis that areas with steeper slopes, and thus, greater exposure to solar wind, tend to accumulate Li-7 transported by the solar wind.

#### 3.1.1. Aitken Crater

Aitken is a large crater located southeast of Heaviside and north of Van de Graaff. Its floor has experienced resurgences from darker lava flows in the past and features several smaller crater impacts on its interior. It is one of the largest craters in the solar system and has a depth of approximately 13 km and a diameter of 2500 km [32].

**Figure 3:**
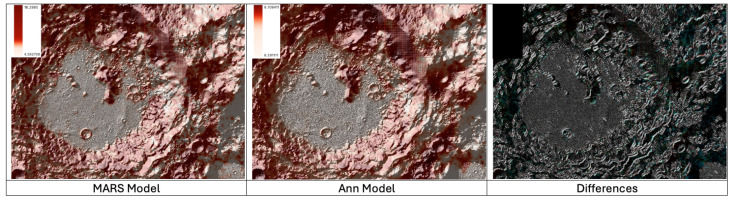
Lithium-7 distribution in Aitken Crater, global visualization of the 2 models used and differences highlighted.

The images in (Figure 3) graphically display the numerical output of the two prediction algorithms developed in the study (MARS and ANN). Darker areas in the images indicate higher predicted concentrations of Li-7, while lighter areas indicate moderate to near-zero predicted values, depending on their intensity.

Additionally, in both predictions, it can be observed that the crater exhibits a substrate of lighter colors, which suggests that the regolith has been removed due to impacts, thus explaining the lower concentration of Li-7 in those areas.

It is also possible to observe the incipient appearance of areas with slightly higher concentrations of Li-7 on the southern walls of the crater, which have a gentler slope and are more exposed to the solar wind compared to flat regions.

It is also worth noting that the MARS model generates maximum Li-7 concentration values significantly higher than those produced by its counterpart ANN. This discrepancy can be attributed to the inherent structure of the model, which relies on a linear sum of polynomial equations sensitive to divergent input values.

**Figure 4:**
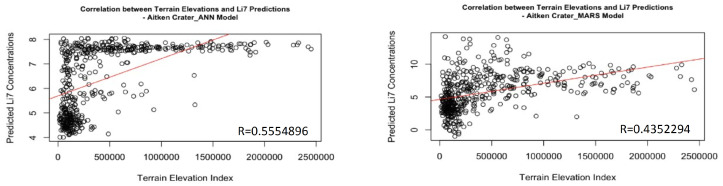
Aitken Crater Correlation of Li-7 Predictions and Terrain Elevation Levels.

Finally, the topographic position index (TPI) [33] values were calculated using QGIS from the available elevation model in ASTROPEDIA, obtained from the Kaguya spacecraft of the Japanese space agency. TPI was computed to extract slope values for each pixel, which are correlated with the Li-7 concentration values obtained for each pixel. The results of the obtained correlation coefficients between Li-7 concentration and elevation index can be observed in Figure 4.

A correlation coefficient of 0.5350985 recorded at Aitken Crater with the MARS model suggests a moderate correlation between Li7 concentrations and measured slopes. This means that there is a discernible relationship between these two variables, but it may not be extremely strong. It is important to remember that correlation does not imply causation; That is, although these two variables are correlated, one does not necessarily cause the other.

As can be seen, there is a clear trend of increasing Li7 concentrations as the elevations of the terrain increase. This relationship is most clearly visualized in Figure 4, which also shows the scatter plot of Li7 concentrations versus slopes, showing a positive correlation between these two variables, suggesting a possible influence of elevation on predicted Li7 concentrations.

#### 3.1.2. Rima Hadley

The Rima Hadley, located on the northeastern rim of the lunar Mare Imbrium, has been a site of particular interest due to its geological significance and its role in lunar exploration. Initially explored during the Apollo 15 mission in 1971, the lunar astronauts David Scott and James Irwin conducted moonwalks in this region, allowing for a detailed study and collection of lunar samples.

Spanning approximately 1.5 km in length and with an average width of around 300 m, covering an area of approximately 450 square kilometers, Rima Hadley is characterized by its steep walls and relatively flat floor. This article highlights its importance not only for its geological insights but also for its historical significance as the site where the lunar roving vehicle (LRV), the first vehicle used on the Moon, was deployed, enabling astronauts to explore a broader lunar landscape and collect samples from various areas [34].

**Figure 5:**
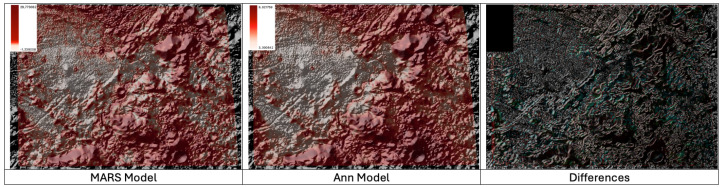
Lithium-7 distribution in Rima Hadley, global visualization of the 2 models used and differences highlighted.

Rima Hadley’s geological significance lies in its exposure of lunar basaltic materials and its contribution to our understanding of the lunar volcanic history. Furthermore, this study explores the concentration of Li-7 within the region. Analysis employing different algorithms reveals slight variations in the central topography, with the MARS model indicating higher Li-7 concentrations compared to the ANN model. Nevertheless, a consistent pattern emerges across all models, supporting the hypothesis that areas with steeper slopes and inclinations exhibit higher Li-7 concentrations in contrast to the relatively flat floor of the rima. The variations in the range of predicted values may account for subtle differences between the models. These findings underscore the importance of predictive studies and contribute to our comprehensive understanding of the lunar landscape and its geological significance. (See Figure 5).

**Figure 6:**
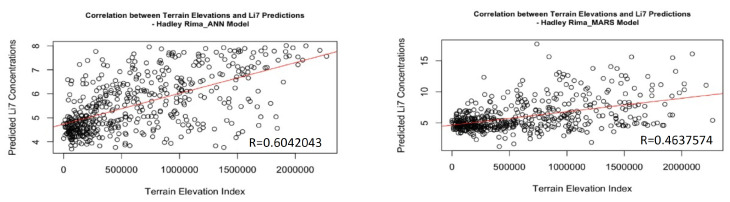
Hadley Rima Correlation of Li-7 Predictions and Terrain Elevation Levels.

The correlation value of 0.4637574 obtained with the MARS model in the case of the Hadley Rima indicates a moderate but weaker correlation between the two variables compared to the previous values. (See Figure 6).

Lunar rilles are linear geological structures on the satellite’s surface. They are characterized by a narrow, winding valley with a much gentler slope compared to craters. This simplicity in their morphology may favor the ANN model due to its greater flexibility and ability to capture complex and non-linear patterns. As a result, this could lead to a more robust correlation with Li7 concentrations in lunar rilles compared to the MARS model, which may have limitations in addressing geomorphological variability.

#### 3.1.3. Apollo 17 Landing In The Taurus–Littrow Valley

The Apollo 17 landing in the Taurus–Littrow Valley not only marked a historic milestone but also a significant scientific achievement. During the mission, astronauts carried out a variety of scientific activities, including drilling the lunar soil, collecting rock and regolith samples, and installing scientific instruments to analyze the composition and properties of the lunar soil. Additionally, detailed observations were made, and photographs were taken of the region [35].

This landing provided valuable information about the geological history of the Moon and contributed to confirming the theory of past volcanic activity on the satellite. The samples and data collected during the mission continue to be the subject of study and analysis by scientists, providing a deeper understanding of the Moon and its evolution.

**Figure 7:**
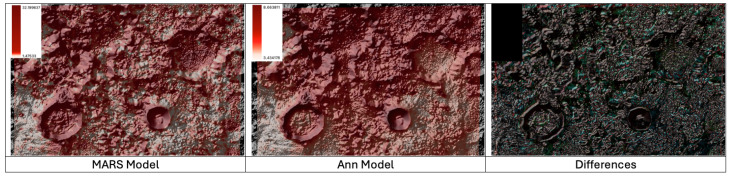
Lithium-7 Distribution in landsite, global visualization of the 2 models used and differences highlighted.

In Figure 7, the noted discrepancies among the two models are observed, with consistency maintained in the steeper areas while varying the results in the southeastern sector. These differences can be justified, as in previous cases, by variations in the predicted maximum and minimum values.

Unfortunately, it was not possible to extract the specific coordinates of the Apollo 17 mission’s landing site from the cartography provided by the Clementine mission. Consequently, it was not possible to confirm the exact values of Li-7 concentration at that specific location. However, it is possible to confirm that the range of values presented by the ANN model across the entire cartography is consistent with expectations. On the other hand, the MARS model continues to show discrepancies, assigning its highest values to more rugged areas exposed to solar wind. It is important to note that these locations are not suitable for the mentioned lunar landing and, therefore, do not represent the actual measurements considered.

**Figure 8:**
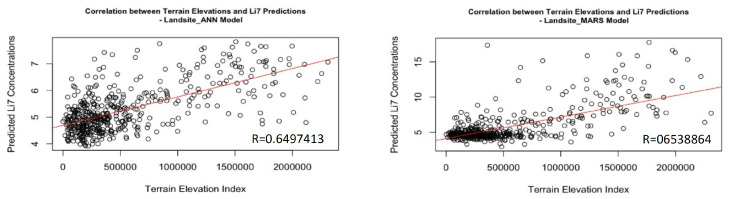
Landsite Correlation of Li-7 Predictions and Terrain Elevation Levels.

The correlation coefficients between 0.5614833 (ANN model for Aitken Crater) and 0.6569838 (MARS model for the landsite) indicate a stronger correlation between Li7 concentrations and slopes, probably due to being an area from which some of the samples used for the modeling of the concentrations of the element in the different areas of the Moon were obtained, suggesting that there is a clearer and more direct relationship between these two variables and strengthening the probability that slopes are contributing significantly to the observed differences in the Li-7 concentrations obtained in each study area. (See Figure 8).

### 3.2. Potential Terrestrial Comparisons and Study Limitations

Incorporating terrestrial data has the potential to enhance the robustness of the findings by offering comparative insights. Specifically, testing models with lithium deposits in established terrestrial locations could provide valuable validation opportunities. However, significant challenges arise due to fundamental disparities between lunar and terrestrial environments. These challenges include differences in atmospheric conditions, geological processes, and the origins of lithium.

The Moon’s lack of an atmosphere allows its surface to be directly exposed to the solar wind, which implants lithium into the lunar regolith. In contrast, Earth’s dense atmosphere prevents solar wind from reaching the surface, necessitating that terrestrial lithium originates from internal geological processes.

On the Moon, lithium accumulates through direct exposure to space and micrometeorite impacts, especially in rugged areas like crater rims. In contrast, Earth’s active geology involves tectonic, volcanic, hydrothermal, and biological processes. Terrestrial lithium occurs in minerals such as spodumene and lepidolite and in evaporitic deposits like salt flats, formed through processes not present on the Moon.

Lunar lithium has accumulated over millions of years via solar wind implantation, a process unique to atmosphereless bodies. Terrestrial lithium, however, forms through geogenetic processes such as high-temperature and high-pressure conditions, hydrothermal activities, and evaporation in salt flats. Additionally, terrestrial lithium is predominantly found within the Earth’s crust, requiring extraction from minerals or brine deposits, unlike the surface-exposed lithium on the Moon.

Given these atmospheric, geological, and origin differences, a comparative study on Earth would not provide meaningful insights into lunar lithium data. The Moon’s unique conditions enabling direct solar wind implantation of lithium are not replicated on Earth.

## 4. Conclusions

In the context of this research, various aspects related to statistical analysis, sample quality validation, resampling, and bootstrapping have been addressed. Additionally, supervised machine learning model training and validation, as well as data import and export, were explored. Multiple tasks were carried out in the QGIS application to enhance our understanding of geographic information systems. This included the import and manipulation of both raster and vector cartographic data, georeferencing, and cartographic information extraction.

Access to various NASA databases allowed for the retrieval of the cartographic information used in this study. By analyzing the data generated by the Clementine probe in the near-infrared (NIR) and ultraviolet-visible (UVVIS) spectra, evidence of the presence of lithium-7 (Li-7) on the lunar surface was detected. These findings support the previous research and significantly contribute to the scientific understanding of the chemical composition of our natural satellite.

It is worth noting that the distribution of Li-7 on the lunar surface is not uniform, and spatial distribution patterns with varying concentrations in different regions of the Moon have been identified. This analysis has allowed for a visual examination of the initial hypothesis that associates surface Li-7 concentration with exposure to solar wind.

However, it is essential to note that a direct numerical relationship between lunar topography and Li-7 concentration has not been established. This is due to the extensive morphological diversity of the study areas and inherent limitations in available methods for extracting information based on topographic indices and other aspects provided by QGIS.

Despite these limitations, preliminary results suggest that the presence of Li-7 on the Moon could have significant economic and technological potential. As Li-7 is a valuable element in various applications, such as high-energy batteries and nuclear technologies, lunar lithium exploration and extraction may present exciting prospects for the future of space exploration.

This work underscores the importance of spatial cartography and remote sensing technology in the study of the Moon and other celestial bodies. The use of data from the Clementine probe in the NIR and UVVIS has allowed for detailed information about lunar chemical composition, offering significant opportunities for future research and space missions that can deepen our understanding of the Moon and other celestial worlds.

It would be highly relevant to conduct a thorough analysis of variations in the distribution of Li-7 on the lunar surface, leveraging the incorporation of data from missions after the Clementine mission, such as the lunar reconnaissance orbiter (LRO). This approach would allow for a deeper understanding of distribution patterns and the potential geological factors influencing these patterns. Furthermore, it would be essential to explore the interplay between the distribution of Li-7 and other lunar features, including mineralogy, topography, and the presence of other elements or compounds. This analysis would shed light on the geological processes and chemical interactions underpinning the observed distribution of Li-7 on the lunar surface.

Moreover, expanding the research to encompass the analysis of the composition of other elements present on the Moon would provide a more comprehensive view of lunar chemistry, enabling a deeper understanding of its implications for the formation and evolution of the solar system.

In the realm of technological and economic applications of lunar Li-7, it would be pivotal to conduct a meticulous analysis of the current trends and demands in related industries such as renewable energy, high-performance batteries, and space technology. This analysis would be crucial for evaluating the commercial potential and future opportunities associated with lunar lithium extraction. Integrating these approaches into the research would represent a significant step towards a more profound understanding of lunar chemistry and its multidisciplinary implications.

Furthermore, in light of the potential applications of the study’s findings in lunar resource exploration, the future research could involve expanding sample collection to encompass a broader range of lunar sites and geological structures, contingent upon new missions landing on the Moon. This expansion would facilitate a more comprehensive understanding of lithium-7 distribution patterns across diverse lunar regions. Moreover, leveraging new remote sensing data acquired through upcoming missions would enhance our ability to map lithium-7 concentrations, thereby advancing lunar resource exploration. Additionally, the incorporation of advanced analytical techniques, such as mass spectrometry, elemental mapping, and isotopic analysis, could provide detailed insights into lithium-7 distribution patterns at the microscale, potentially enabling researchers to characterize mineralogical compositions and identify geological processes that influence lithium-7 distribution across different lunar regions.

## Figures and Tables

**Table 1:** Descriptive statistics of the original sample: Concentration of lithium-7 (ppm) and reflectance values (unitless) at specific ultraviolet and visible spectrum wavelengths (micrometers).

	LithiumD7	UVV-415	UVV-750	UVV-900	UVV-950	UVV-1000
Minimum	3.350	0.06720	0.1079	0.1093	0.1104	0.1140
Median	4.375	0.07350	0.1247	0.1259	0.1286	0.1322
Mean	4.379	0.08254	0.1351	0.1399	0.1418	0.1467
Maximum	8.890	0.12844	0.2054	0.2175	0.2205	0.2269

**Table 2:** Descriptive statistics of the original sample: Reflectance values (unitless) at specific near-infrared spectrum wavelengths (micrometers).

	Nir1-1100	Nir2-1250	Nir3-1500	Nir4-2000	Nir5-2600	Nir6-2780
Minimum	0.1118	0.1247	0.1314	0.1511	0.2276	0.7467
Median	0.1474	0.1620	0.1777	0.2281	0.3421	0.9277
Mean	0.1534	0.1673	0.1848	0.2271	0.3611	0.9394
Maximum	0.2303	0.2504	0.2745	0.3151	0.4909	1.1517

**Table 3:** Descriptive statistics of the original sample: Concentration of lithium-7 (ppm) and reflectance values (unitless) at specific ultraviolet and visible spectrum wavelengths (micrometers).

	LithiumD7	UVV-415	UVV-750	UVV-900	UVV-950	UVV-1000
Minimum	3.919	0.07126	0.1171	0.1208	0.1221	0.1268
Median	4.882	0.08195	0.1334	0.1377	0.1401	0.1443
Mean	4.959	0.08262	0.1352	0.1399	0.1419	0.1467
Maximum	6.436	0.10386	0.1659	0.1739	0.1772	0.1818

**Table 4:** Descriptive statistics of the sample generated by bootstrap: Reflectance values (unitless) at specific near-infrared spectrum wavelengths (micrometers).

	Nir1-1100	Nir2-1250	Nir3-1500	Nir4-2000	Nir5-2600	Nir6-2780
Minimum	0.1197	0.1324	0.1402	0.1650	0.2475	0.7835
Median	0.1538	0.1673	0.1859	0.2293	0.3654	0.9468
Mean	0.1537	0.1676	0.1851	0.2278	0.3604	0.9391
Maximum	0.1878	0.2028	0.2282	0.2785	0.4465	1.0542

## Data Availability

The data are contained within the article.
